# A threshold explanation for the lack of variation in negative composite time trade-off values

**DOI:** 10.1007/s11136-022-03155-6

**Published:** 2022-05-27

**Authors:** Bram Roudijk, Rogier Donders, Peep Stalmeier

**Affiliations:** 1 EuroQol Research Foundation, Rotterdam, The Netherlands; 2 Radboud University Medical Center, Radboud Institute for Health Sciences, Nijmegen, The Netherlands

**Keywords:** Composite time trade-off, cTTO, Utilities, Negative values

## Abstract

**Background:**

Recent studies concluded that for health states considered worse than dead (WTD), as measured with the time trade-off (cTTO) method, negative mean values were insensitive to health state severity, which represents a validity problem for the cTTO. However, the aforementioned studies analysed negative values in isolation, which causes selection bias as the value distribution is truncated.

**Aim:**

To investigate the validity of aforementioned studies and of negative values in general.

**Methods:**

The ‘threshold explanation’ was formulated: beyond a certain severity threshold, preferences change from better than dead (BTD) to WTD. This threshold differs between respondents. Thus, negative values across severity are obtained from different respondents, and responses added for higher severity contribute negative values close to zero, explaining the aforementioned insensitivity. This explanation was tested using data from the Dutch EQ-5D-5L valuation study. Respondents valued 10 health states. Based on respondents’ number of WTD preferences, segments were constructed, containing respondents with similar severity thresholds. Using regression models for each individual respondent, we examined the relation between values and severity and compared respondents between segments.

**Results:**

Negative values, when analysed in isolation, were insensitive to severity. However, for individual respondents and within most segments, cTTO values and severity were negatively related. For individual respondents, negative slopes were steeper for segments with more WTD preferences, as predicted by the threshold explanation.

**Discussion:**

Analysing negative values in isolation leads to biased estimates. Analyses of cTTO values for individual respondents refute the insensitivity of negative cTTO values.

**Supplementary Information:**

The online version contains supplementary material available at 10.1007/s11136-022-03155-6.

## Introduction

Health utilities are commonly used in economic evaluations in health care. Generic preference-based instruments such as the EQ-5D allow for the comparison of different diseases on a single scale, anchored on 0 (dead) to 1 (full health), whilst health states worse than dead (WTD) are assigned negative values [1–3]. To measure health utilities, various valuation methods such as the standard gamble, time trade-off (TTO), Visual Analogue Scale (VAS), composite time trade-off (cTTO), and discrete choice experiments (DCE) are used [4–6]. In the TTO method, respondents are asked to value health states by choosing between two different hypothetical lives, one of which is being for a varying number of years in full health and the other being for a fixed duration in a certain impaired health state. The duration of life in full health is varied until indifference is reached. For example, if a respondent is indifferent between living for 6 years in full health or for 10 years with chronic back pain, the health state “chronic backpain” is valued at 0.6 under the assumptions of the QALY model. The cTTO is a variation of the TTO method intended to reduce the cognitive burden of health states considered WTD and produces values on a scale from − 1 to 1.

The cTTO and TTO methods face an important problem: insensitivity to the severity of WTD states, that is, for states WTD, negative values do not vary much [7–9]. Here, severity is calculated as the sum of the levels on the EQ-5D dimensions of the health state. Gandhi et al. used regression analyses to show that the mean value for WTD states does not change over severity [8]. In contrast, for valuations of health states considered better than dead (BTD), that is, with positive values, there was a reliable negative relation between values and severity. In previous studies using TTO with a different method to value WTD states as compared to the cTTO, similar observations were made [7, 10]. Busschbach et al. attribute this to the task difficulty of the WTD task in the TTO [7, 10]. As one may expect to find lower negative health state valuations for more severe health states, the insensitivity questions the validity of the WTD part of the cTTO or TTO tasks. However, the distribution of values is truncated to either positive or negative values. As shown by Hausman and Wise, estimates based on truncated value distributions are biased [11]. As respondents differ in whether they consider any health state as WTD or which states they consider WTD, truncating the value distribution to either positive or negative values effectively causes selection bias.

Although previous authors have stated that analysing BTD or WTD preferences separately is not a correct approach, no clear link to truncated samples causing selection bias was made previously [12]. Our study takes this selection bias into account and proposes a possible explanation for the above lack of sensitivity from a *preference* point of view, which we will call the *threshold explanation*, further explained in the methods section. In valuation studies, respondents value health states of varying severity, usually around 10 per person. As the severity of these health states increases, more respondents will have negative values, whereas fewer respondents have positive values. Beyond a certain severity threshold, preferences change from BTD to WTD. This threshold where BTD responses change into WTD preferences differs between respondents. Thus across severity, the samples of respondents change when positive and negative values are analysed in separation, leading to truncated distributions within and across respondents. Our aim is to show that such a change in samples explains the alleged lack of sensitivity for negative values.

## Methods

### Previous work and the threshold explanation

In a previous study, Gandhi et al. observed the aforementioned insensitivity to severity through an analysis of data collected in several national valuation studies for the EQ-5D-5L instrument [8]. In these valuation studies, cTTO data are collected in a large representative sample of around 1000 respondents, each valuing 10 health states using the cTTO method [13, 14]. The authors then conducted regression analyses on the negative values and positive values separately, regressing the values on the accompanying severity of the valued health states. Severity is defined as how far the valued health state differs from full health, with larger deviations being considered more severe. This was done separately for each national dataset, along with additional analyses to ensure robustness.

Each of these analyses resulted in a negative relation between values and severity for the positive values, and no significant relation between values and severity for the negative values. In a similar analysis, Busschbach et al. rank the observed mean values for EQ-5D-3L health states collected in three countries, for positive and negative values separately [7]. These data were collected using the TTO method, as used in the Measurement and Valuation of Health study (MVH), the first national value set study for the EQ-5D instrument [10]. Busschbach et al. also observed that the mean values observed for each health state differ much more for positive values when compared to negative values.

**Fig. 1:**
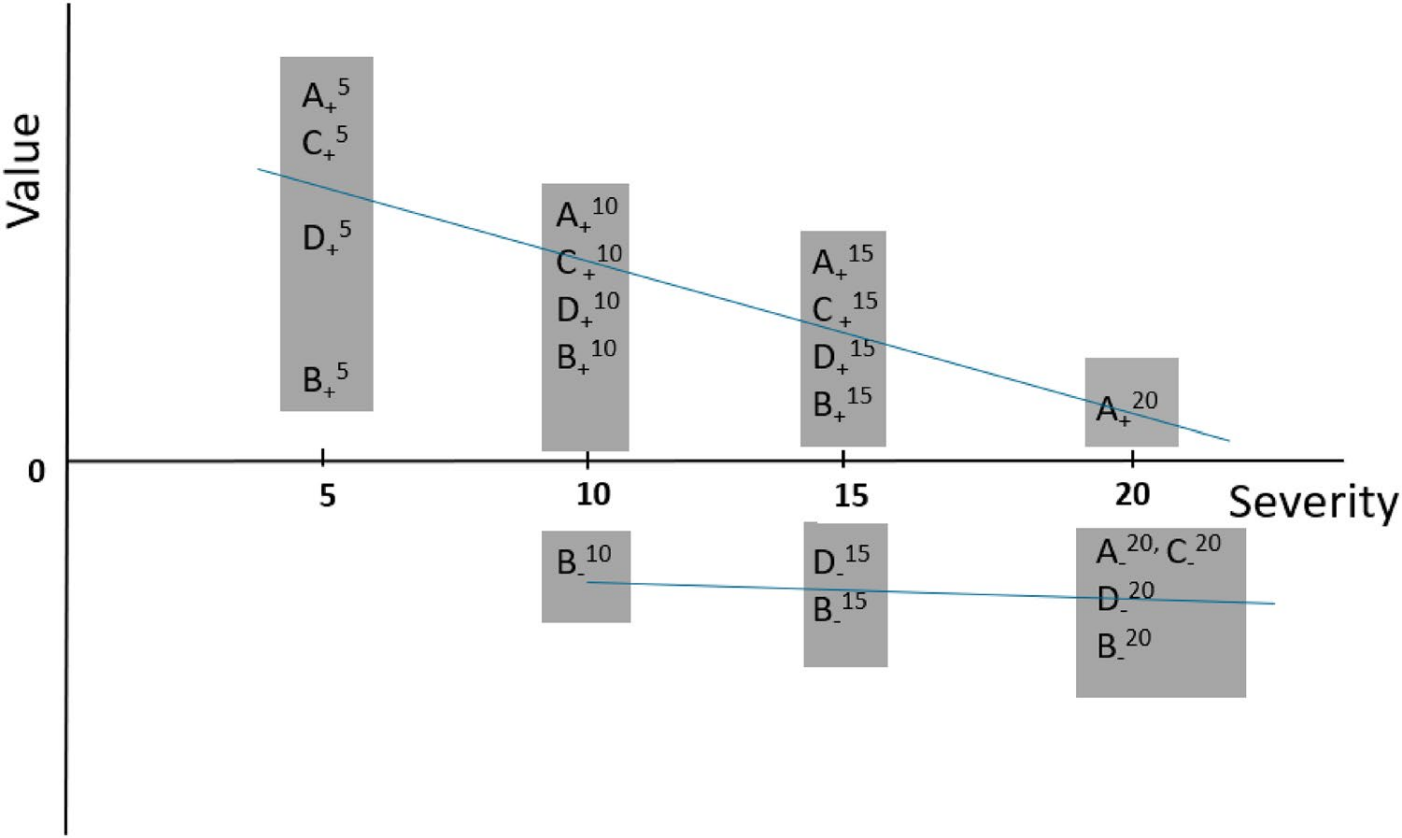
Illustration of the change in samples across severity. Figure explained in the main text

However, Hausman and Wise showed that truncations based on the dependent variable, here positive versus negative values, lead to biased estimates: “uses of the data that treat components of …values… as dependent variables in a least squares regression framework will lead in general to parameter estimates that are biased in a predictable direction and inconsistent” [11]. Figure 1 illustrates the situation of truncated samples for health valuations. Values for states with severity 5, 10, 15, and 20 are plotted for respondents A, B, C, and D. For the health state with severity 5, all respondents give positive values. For health states with severity 10, 15, and 20, an increasing number of negative values occur. However, these negative values originate from different respondents: e.g. for severity 10, only respondent B contributes to the negative values, for severity 15, D and B contribute, and for severity 20, all respondents contribute. Of note, across severity, respondents are added with negative values close to zero: e.g. for severity 15, Fig. 1, negative values close to zero of respondent D are added, and for severity 20, negative values close to 0 of respondents A and C are added. This will keep the sample means of negative values relatively stable across severity, and consequently, the slope is biased upwards. In terms of regression analyses, we have ${V}_{ij}= {\beta }_{\mathrm{i}}*{S}_{ij}+\varepsilon ij$, where ${V}_{ij}$ represents the value assigned to a health state (range − 1 to 1) and ${S}_{ij}$ represents the severity (range 0 to 20) of health state *j, j* = 1,2,…10, valued by respondent $i$. ${\beta }_{\mathrm{i}}$ represents the slope for respondent *i* and ${\varepsilon }_{ij}$ is the error term (ranges are explained below). When analysing the responses for positive and negative values in separation, values for health states of each respondent are split between positive and negative values. As explained in Fig. 1, for negative values, only a subset of respondents *i* contribute an observation of ${V}_{ij}$ for ${S}_{i}$, and moreover this subset of respondents *i* changes across severity. In other words, valuations of all health states by all respondents are not observed when the sample is stratified and separately analysed for WTD and BTD preferences, which effectively leads to selection bias. This will lead to biased estimates for ${\beta }_{\mathrm{i}}$ for WTD preferences and *mutatis mutandis*, also for BTD preferences [11]. We therefore conclude that previous regression analyses on negative values suffer from selection bias. Henceforth, we will call the previous analyses on negative values in isolation the *WTD-insensitive* explanation, as previous authors concluded that WTD preferences were insensitive towards severity.

We now present an alternative explanation, in which it is assumed that respondents are heterogeneous in their preferences. When respondents value a number of health states, some respondents consider certain health states as WTD and other respondents will not. After a certain “threshold” of severity, respondents are more likely to consider a health state as WTD. For example, in Fig. 1, for respondent B, the threshold is somewhere between 5 and 10, whereas for respondent A, between 15 and 20. Values for states more severe than the threshold are more likely to be negative. Since the severity threshold differs between respondents, the samples of negative values contain an increasing number of respondents across the severity, agreeing with the reasoning above based on Fig. 1 in the previous paragraph. Henceforth, we will call this the *threshold explanation*.

**Fig. 2:**
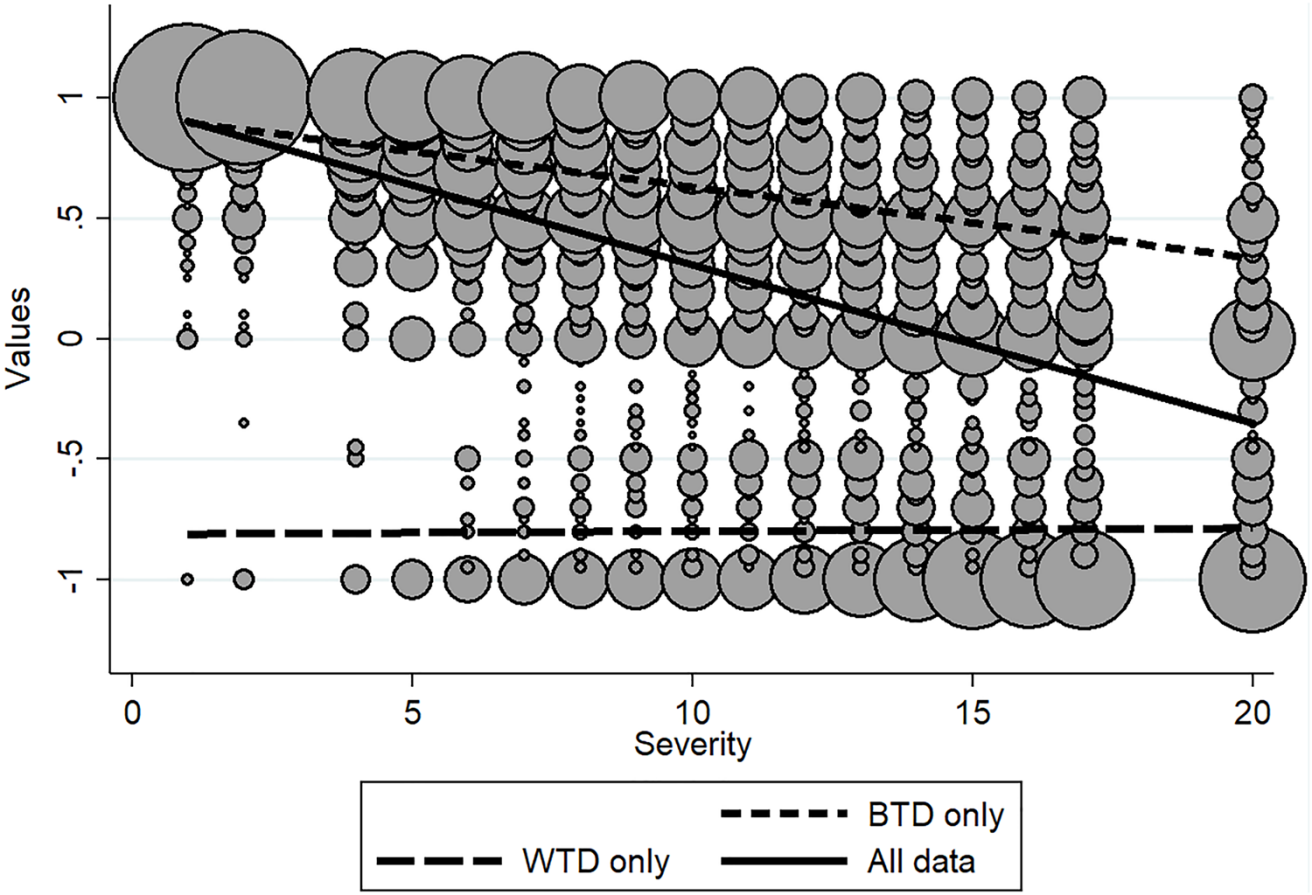
Distribution plot of the reported values by the severity of those health states (*x* axis). Larger circles indicate a larger number of observations for that value/severity combination. Fit plots are included for the BTD data only (upper-stippled line), WTD data only (lower-stippled line), and BTD and WTD data combined (solid line). The circles are scaled based on the number of responses given for a certain severity

To avoid using truncated samples, preferences can be analysed at the respondent level, combining their BTD preferences and WTD preferences in the same analysis. Then, different predictions can be derived from the WTD-insensitive and threshold explanations. These predictions pertain to the intercepts and slopes of the regression lines of severity on values and use the experimental observation that for severity 1, almost all values are concentrated at 1 (see Fig. 2).

Regarding the intercepts, one may expect that respondents with more WTD preferences, and thus more negative values, will have a lower regression *intercept*. However, it is not clear how predictions for the *intercepts* differ between the WTD-insensitive and threshold explanations. Predictions regarding the *slopes*, however, differ considerably between the WTD-insensitive and threshold explanations. For the WTD-insensitive explanation, the separate analyses on truncated negative values report a correlation between negative values and severity of only 0.015 [8], suggesting that negative values amount to noise. Therefore, the WTD-insensitive explanation predicts shallower negative slopes for respondents with a larger number of WTD preferences. More specifically, if the WTD-insensitive explanation is valid, individual respondents are not expected to exhibit slopes smaller than zero for negative values.

The threshold explanation, however, makes a different prediction. For the respondents with more WTD preferences, the threshold moves to the left to accommodate the higher number of WTD preferences. Given that values are concentrated at 1, steeper negative slopes are expected for segments with more negative values. In conclusion, whereas the WTD-insensitive explanation predicts shallower negative slopes, the threshold explanation predicts steeper negative slopes for respondents with a larger number of worse than dead preferences.

### Data

Existing data from the Dutch EQ-5D-5L valuation study data were used to test our hypothesis that changing samples cause the alleged insensitivity of values for states WTD to severity [15]. In this study, a sample of 983 respondents from the general public completed 10 cTTO tasks. For a detailed description of the sample, we refer to Versteegh et al. [15]. More details on the EQ-5D-5L instrument and the cTTO method are presented in the appendix or can be read elsewhere [13, 14, 16] (also see online appendix). The Dutch EQ-5D-5L study used the standard EQ-VT design of 86 health states, out of which each respondent values 10 health states [17]. The health state design comprises 80 health states that are selected to be optimally suitable to be used for main effects regression analyses in valuation studies and is supplemented by 6 additional health states. These are the very mild states where there is only one deviation from full health, and the worst health state described by the EQ-5D-5L instrument, in which one has extreme problems on all 5 EQ-5D-5L dimensions. The 86 health states were divided in 10 blocks on 10 health states, in which each block is assigned 1 of the 5 mild states, the health state 55,555, and 8 states from the optimal design. Utility balance was used as a criterion for the blocking of health states, which means that although the between-block variance is minimized the severity will differ between the blocks [17]. The severity of a health state is operationalized as the sum of all the number of deviations from full health [8]. Here we count the levels deviations, minus 5. As the EQ-5D-5L has 5 dimensions and five levels, this score is 0 as a minimum and 20 as a maximum. For example, a health state in which one has level 2 problems (1 deviation from full health) on one dimension, level 3 problems on another (2 deviations), level 5 problems on one dimension (4 deviations), and no deviations from full health one two dimensions, the severity score would be 1 + 1 + 2 + 3 + 5–5 = 7.

### Analyses

#### Replication of previously reported analyses

Regression fit plots will be used to determine whether the previously reported insensitivity towards severity for states WTD can be replicated [8]. In addition, distribution plots are used to further explore the distribution of values over the severity domain.

#### OLS models for individual respondents

Each respondent valued 10 health states through cTTO, of differing severity. For each respondent, we can then separately estimate a simple OLS regression analysis to determine the relation between value and severity, as described in Eq. 1:

1 $${V}_{ij}={\beta }_{0j}+{\beta }_{1j}\times {S}_{ij}+{\varepsilon }_{ij}.$$

Here ${V}_{ij}$ represents the value $V$ assigned to health state $i$, by respondent $j$. ${S}_{ij}$ represents the severity $S$ of health state $i$ as valued by respondent $j$. ${\beta }_{0j}$ represents the regression intercept for respondent $j$, whilst ${\beta }_{1j}$ represents the regression slope for severity for respondent $j$. ${\varepsilon }_{ij}$ represents the error term, which we assume to be distributed as ${\varepsilon }_{ij}\sim N(0,{\sigma }_{{\varepsilon }_{ij}}^{2})$.

In addition, we will estimate a regression model for each respondent of the form of Eq. 2:

2 $${V}_{ij}={\beta }_{0j}+{\beta }_{1j}\times {S}_{ij}+{\beta }_{2j}\times {S}_{ij}\times {\mathrm{WTD}}_{ij}+{\varepsilon }_{ij}.$$

This is the same model as in Eq. 1, but with the addition of the term ${\beta }_{2j}\times {S}_{ij}\times {\mathrm{WTD}}_{ij}$, which represents an interaction between severity (${S}_{ij}$) and whether health state $i$ is considered better or worse than dead by respondent $j$. ${\mathrm{WTD}}_{ij}$ is a dummy variable coded as 0 (BTD) or 1 (WTD). Equation 2 essentially provides a slope for severity for positive values, ${\beta }_{1j}$, and a slope for severity for negative values, ${\beta }_{2j}$, for every respondent $j$. Thus it can be observed whether the slopes for negative values differ significantly from 0, and, in addition, it can test whether the slopes for positive and negative values differ on average, using an unpaired t test. Note that in Eq. 2, we are not truncating distributions, thus avoiding selection bias.

#### Segmenting respondents by their number of negative values

Respondents are segmented into $k$ groups, based on the number of health states $k$ they considered WTD in the cTTO task. As each respondent valued 10 health states, $k$ ranges from 0 to 10. For segments for which $k$ is small, the low number of WTD states corresponds with a severity threshold that is more to the right as explained above; vice versa, for segments with higher $k$, the threshold will be more to the left. Using scatterplots, the slope ${\beta }_{1j}$ (vertical axis) is plotted against the intercept ${\beta }_{0j}$ (horizontal axis), for each individual respondent. Similar plots are made for the models estimated in Eq. 2, where the slopes, not intercepts, are split into ${\beta }_{1j}$ for positive values and ${\beta }_{2j}$ for negative values. In addition, the median slopes and median intercepts are estimated within segments.

## Results

### Replication of previously reported analyses

Figure 2 shows the distribution of the values by severity, where larger dots indicate a larger number of values for that particular value/severity combination. This figure contains fit plots to represent the relation between severity and values in the BTD only, WTD only, and positive and negative values combined. The WTD line is horizontal, replicating the previously reported insensitivity towards severity [8].

### OLS models for individual respondents

**Fig. 3:**
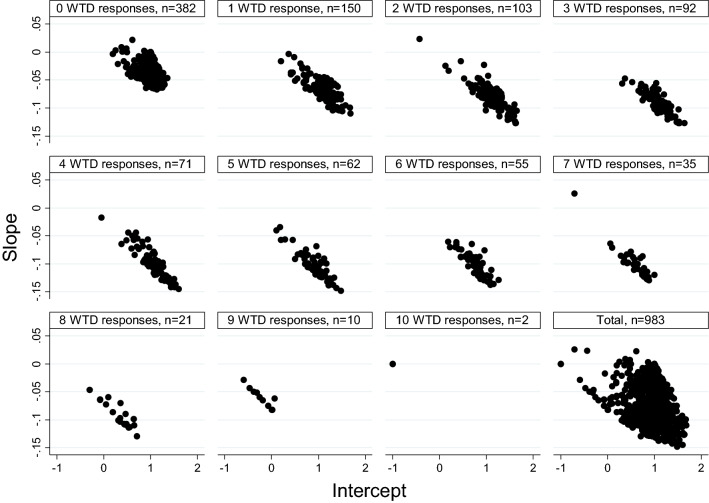
Slopes (vertical axis) and intercepts (horizontal axis) for the relation between values and severity for each respondent. Respondents are segmented into groups by their number of WTD preferences, ranging between 0 and 10. Each point in these scatter plots represents the slope and intercept for an individual respondent. The final panel represents the aggregate data

We now turn to the analyses of the relation between values and severity at the *individual* level using OLS regression (Eq. 1). The scatter plots in Fig. 3 show the *slopes* (vertical axis) and *intercepts* (horizontal axis) for values and severity for each respondent. The respondents are segmented into groups based on the number of WTD preferences. Table 1 shows the sample size, sample means for cTTO values, and 95% confidence intervals of the mean cTTO values for each segment. The top left panel in Fig. 3 shows the slopes and intercepts for respondents without WTD preferences, whilst the next panel shows the slopes and intercepts for respondents that provided one WTD preference and so forth. The final panel combines the respondents from all segments. These plots show that respondents are relatively homogeneous in their slopes and intercepts within segments, more than they are between segments as shown in panel 12. For *k* = 0, the slope (vertical axis) is about, − 0.05, as expected since values drop from 1 to zero, whilst the health state severity ranges from 1 to 20.

**Table 1:** Sample size per segment and mean cTTO value

Segment/number of WTD responses	Number of respondents	Mean cTTO value	95% CI mean cTTO values
0	382	0.641	[0.630, 0.651]
1	150	0.445	[0.421, 0.470]
2	103	0.318	[0.281, 0.355]
3	92	0.198	[0.154, 0.242]
4	71	0.075	[0.019, 0.131]
5	62	− 0.099	[− 0.160, − 0.038]
6	55	− 0.240	[− 0.304, − 0.175]
7	35	− 0.406	[− 0.488, − 0.325]
8	21	− 0.585	[− 0.678, − 0.492]
9	10	− 0.810	[− 0.907, − 0.712]
10	2	− 1	[− 1.000, − 1.000]
Total	983	0.317	[0.304, 0.330]

**Fig. 4:**
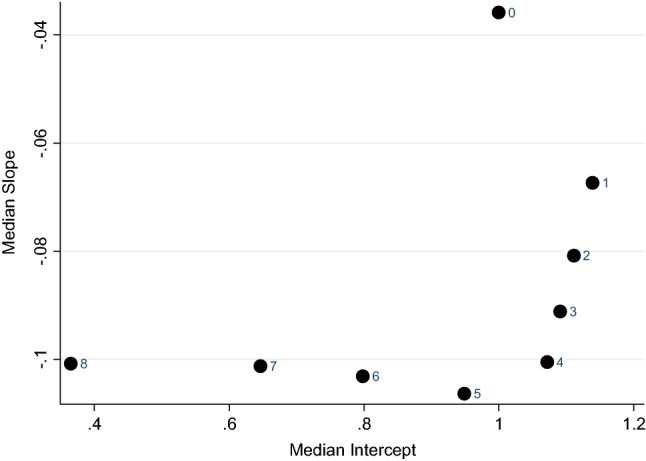
Median slopes (vertical axis) and intercepts (horizontal axis) for each segment. The markers indicate the number of WTD preferences in each segment

A visual inspection of these plots shows that the point clouds move downwards, that is, the individually estimated slopes (see vertical axis) become more negative for segments with more WTD preferences. The individually estimated intercepts remain about the same. This is illustrated in Fig. 4, where the median slopes and intercepts are presented for each segment, based on the data in Fig. 3. As shown in Fig. 4, across segments, the median slope decreases, whilst the median intercept remains similar at first and then drops for the higher segments. The reduction in slope is consistent with the threshold explanation, but inconsistent with the WTD-insensitive explanation. Segments with more than 7 WTD preferences exhibited a floor effect and contained few respondents, that is, 21, 10, and 2 for segments 8, 9, and 10, respectively, amounting to 3% of the total sample. These segments are therefore not shown in Fig. 4.

**Fig. 5:**
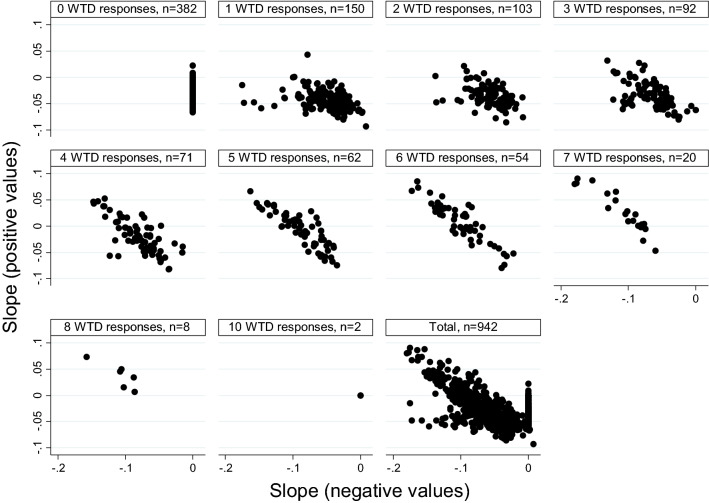
Slopes for *positive values* (vertical axis) and slopes for *negative values* (horizontal axis) for the relation between values and severity for each respondent (see Eq. 2). Respondents are segmented into groups by their number of WTD preferences, ranging between 0 and 10. Each point in these scatter plots represents the slopes for positive and negative values for an individual respondent. The final panel represents the aggregate data. Respondents with a slope larger than 0.1 for positive values were omitted (*n* = 41). In the first panel, 0 WTD responses, the slope for negative values is undefined, as respondents do not exhibit WTD preferences. Similarly in panel 10, where all responses are negative, the slope for positive values is undefined. We have set these slopes to 0 in Figure, to enable us to present the slope for positive values in the first panel and the slope for negative values in the 10th panel

Further regression analyses at the individual level are explored as described in Eq. 2, in which separate slopes for the positive and negative values were estimated. The results of these analyses are shown in scatterplots in Fig. 5, where *slopes* for positive values are plotted on the vertical axis, and the *slopes* for negative values on the horizontal axis. Note that 41 respondents were excluded to maintain interpretability of the graphs, as they had large positive slopes, that is, larger than 0.1, for positive values. Such positive slopes are inconsistent as negative slopes are expected. These 41 respondents had either 6 (1 respondent), 7 (15 respondents), 8 (15 respondents), or 9 (10 respondents) WTD preferences. We found mean slopes of − 0.028(95% CI [– 0.030, − 0.026]) for positive values and − 0.041(95% CI [− 0.044, − 0.039]) for negative values. An unpaired *t* test showed a significant statistical difference between the two means (*t* = 7.788, *p* < 0.00), with the mean slope for negative values being significantly more negative.

## Discussion

### Main findings

This study replicates findings of previous studies, in which cTTO values were found to be insensitive to the severity of health states when analysing the negative values in isolation [8]. Our threshold explanation holds that samples change across severity, for which previous studies did not account. Our analyses were conducted at the respondent level and showed negative slopes for most respondents. After segmenting respondents by their number of WTD preferences, negative slopes between severity and health values were found in each segment. As predicted by the threshold explanation, the slopes were more negative for respondents in segments with more health states considered to be worse than dead.

### Interpretation

The threshold explanation is supported by Fig. 2. In this figure, almost all values are 1 for severity the lowest severity. But at higher severity, this is no longer the case as more negative values occur. This observation makes immediately clear that the BTD samples are large for less severe states and, vice versa, that the WTD samples are larger for more severe states. When analysing BTD or WTD samples in isolation, estimates are biased [11]. This suggests that the WTD-insensitive explanation needs further scrutiny. To continue, as respondents move past their severity threshold, positive values close to 0 become negative values but are still close to 0. As a result, the increasing frequency of negative values at minus 1 across severity is in part cancelled by new responses close to 0, moving into the negative value range and weakening the value–severity relationship for negative values.

The WTD-insensitive explanation suggests that negative values amount to noise, based on the putative low correlation between negative values and severity. If so, “flatter” or less negative slopes are to be expected for the segments with more negative values. In contrast, the threshold explanation predicts steeper or more negative slopes for segments with more negative values. Segment analyses disconfirmed the WTD-insensitive prediction and instead supported the prediction of the threshold explanation because slopes become more negative for the higher segments. In conclusion, our analyses do not support the conclusion that negative values are invalid. Instead, our analyses show that on average, respondents have negative slopes both for positive and negative values. Surprisingly, the slopes for negative values are even more negative than for positive values.

Using different thresholds than 0 to separate responses yield yet other slopes for values above and below that threshold. For instance, using a threshold of 0.8 results in a slope close to zero for values above the threshold and a slope of − 0.05 below the threshold (data not shown). This exemplifies the problematic nature of analyses on data that are split by a threshold. This reflects the previous explanation “that the lack of separate analyses for positive and negative values may not be surprising given that all states appear in each sample (i.e. every state is rated as better than dead or worse than dead by someone” [12].

Even though our results suggest that negative values are not insensitive, challenges remain for the cTTO method and negative values in general, as negative values are in principle unconstrained. For instance, the bottom value of -1 in the cTTO method depends on the arbitrary choice of the lead time duration. Thus, it is unclear what negative utilities represent. The Better than Dead method may be considered in this discussion, as it constrains values to minus 1 without arbitrary methodological choices [18].

Sample selection bias seems to be a major factor causing the insensitivity towards severity (see Hausman and Wise, page 922, Fig. 1. Hausman and Wise state the following in their paper: *“In addition to this explicit concentration of effort, economic data are often recorded only if they fall within prescribed intervals. For example, the values of inheritances are recorded only if they are over sixty thousand dollars”* [11]. The framework set out by Hausman and Wise may then be applicable to topics in health sciences and health economics as well. An example could be analysing data on patients receiving health care and their income, separating between higher and lower income strata, where strata are defined based on income.

### Limitations and strengths

A limitation of this study is that a single cTTO dataset was used to analyse the insensitivity to severity. Previous studies combined multiple countries’ datasets to analyse this issue, which may lead to more generalizable results than ours. However, the Dutch EQ-5D-5L data were used in the multi-country study as well and was no outlier [8]. Second, secondary data analysis was used for hypothesis testing. Qualitative studies may inform us more about the thought process behind the threshold explanation and which considerations matter for respondents to consider health states WTD [5, 19]. Lastly, a limitation of this study is that we excluded 41 respondents in the analyses described by Eq. 2, which is about 4.2% of the sample. However, for these respondents, a large positive slope was found for their responses for positive values, indicating that their responses were inconsistent, as higher values were assigned to more severe states for BTD responses.

A strength of this study is that all analyses were conducted at the respondent level, rather than the aggregate level. Therefore, subsequent segmentations of the respondents do not suffer from potential selection bias induced by truncating the dependent variable, as all estimations were performed before segmenting respondents. Another strength of this study is that using these segments, we were able to verify testable predictions from the WTD-insensitive and threshold explanations.

## Conclusion

This study refutes previous analyses and findings regarding the insensitivity towards severity for negative values [7, 8]. Our analyses show that slopes for negative values are negative, more so in segments with more WTD preferences. These results support the threshold explanation. Our findings therefore contradict the conclusions of previous studies, in which the WTD task of the cTTO was deemed to be invalid due to the alleged insensitivity towards severity. In conclusion, our findings suggest that negative values are sensitive towards severity, which strengthens the position of the cTTO method as used until now [14, 16].

## Supplementary Information

Below is the link to the electronic supplementary material.

Supplementary file1 (DOCX 20 kb)
