# Supplementary material for: A threshold explanation for the lack of variation in negative composite time trade-off values
Source: Qual Life Res. 2022 May 27;31(9):2753–61. doi: 10.1007/s11136-022-03155-6 (PMC9356942; doi:10.1007/s11136-022-03155-6)
Supplement: Supplementary file 1 — Supplementary file1 (DOCX 20 kb) [file 11136_2022_3155_MOESM1_ESM.docx]

**Appendix**

**Instrument: EQ-5D-5L**

The health utility instrument used in this study is the EQ-5D-5L.[1] The EQ-5D-5L is a health state classification system, consisting of 5 domains; mobility, self-care, doing their usual activities, pain or discomfort and anxiety and depression. Each of these domains have 5 levels of problems: no problems, slight problems, moderate problems, severe problems or are unable to/have extreme problems on that health domain. Combining the scores on each of these 5 domains results in a health profile, to which a numerical identity can be assigned. An example of such an identity would be 14532, indicating that this person has no problems with walking about, has severe problems with washing or dressing, is unable to do their usual activities, has moderate pain or discomfort and is slightly anxious or depressed. The order of the numbers reflects the health domain, and the size of the number reflects the level of the problems, where 1 indicates no problems, and 5 indicates extreme problems on that domain. These health profiles can then be assigned a value using a value set, also called a tariff.

**Valuation method: cTTO**

To generate a tariff, the preferences for health states are measured in a target population, by presenting a sample of that population with a valuation task. cTTO is often used when generating tariffs for the EQ-5D-5L instrument.[5, 14, 15] Each respondent completes multiple cTTO tasks for a set of health states. Using a model, values are then predicted for all possible health states.

The cTTO is an indifference procedure in which respondents can trade hypothetical life years in good health to avoid living in some suboptimal health state for 10 years. The cTTO starts with the question of whether the respondent prefers option A) living for 10 years in good health followed by death, or option B) 10 years in health state Y followed by death. If the respondent prefers living in good health for 10 years, he or she is presented with the choice of dying immediately or living in the health state Y for 10 years. If living in health state Y is preferred, the respondent is presented with the choice between living 5 years in good health or 10 years in the state Y. Based on the previous response, either a year or a half of a year is added or subtracted from the number of life years in good health, until indifference is reached. If the respondent chooses immediate death over 10 years in health state Y, he or she will be presented a new task, in which 10 years in good health precedes the 10 years in the diseased state for option B. The task starts again with option A being 10 years in good health followed by death, but option B now contains 10 years in good health, followed by 10 years in state Y, followed by death. If A is preferred, the next question will be whether the respondent prefers 5 years in good health or option B, with option B remaining the same as in the previous question. This “WTD task” is identical to the “BTD task” in all aspects except the 10 years in full health preceding the 10 years in state Y.

Values for the responses are calculated using the QALY model. For example, if a respondent is indifferent between living for 5 years in good health and 10 years in some health state $Z$, the QALY model shows us that the value assigned to health state Z would be $5*1=10*v(Z)$, so the value for $Z$ would be 0.5. In other words, the values for health states can be found using the following transformation $v\left( Z \right)=(10-x)/10$, where x is the amount of life years traded in the BTD task. 20 years can be traded in the WTD task, which means that the highest value a respondent can assign to a health state is 1, while the lowest is -1.
